# Direct Detection of Heterotrophic Diazotrophs Associated with Planktonic Aggregates

**DOI:** 10.1038/s41598-019-45505-4

**Published:** 2019-06-26

**Authors:** Eyal Geisler, Anne Bogler, Eyal Rahav, Edo Bar-Zeev

**Affiliations:** 1 Zuckerberg Institute for Water Research (ZIWR), The Jacob Blaustein Institutes for Desert Research (BIDR) Ben-Gurion University of the Negev, Sede Boqer Campus, 84990 Israel; 2 Israel Oceanographic and Limnological Research, National Institute of Oceanography, Haifa, 8030 Israel

**Keywords:** Microbial ecology, Water microbiology

## Abstract

N_2_ fixation by planktonic heterotrophic diazotrophs is more wide spread than previously thought, including environments considered “unfavorable” for diazotrophy. These environments include a substantial fraction of the aquatic biosphere such as eutrophic estuaries with high ambient nitrogen concentrations and oxidized aphotic water. Different studies suggested that heterotrophic diazotrophs associated with aggregates may promote N_2_ fixation in such environments. However, this association was never validated directly and relies mainly on indirect relationships and different statistical approaches. Here, we identified, for the first time, a direct link between active heterotrophic diazotrophs and aggregates that comprise polysaccharides. Our new staining method combines fluorescent tagging of active diazotrophs by nitrogenase-immunolabeling, polysaccharides staining by Alcian blue or concanavalin-A, and total bacteria via nucleic-acid staining. Concomitant to N_2_ fixation rates and bacterial activity, this new method provided specific localization of heterotrophic diazotrophs on artificial and natural aggregates. We postulate that the insights gained by this new visualization approach will have a broad significance for future research on the aquatic nitrogen cycle, including environments in which diazotrophy has traditionally been overlooked.

## Introduction

Biological dinitrogen (N_2_) fixation is an important source of new bioavailable nitrogen in many marine and freshwater environments^1,2^. This process is carried out by a specialized subgroup of prokaryotic organisms termed diazotrophs, and primarily hindered by four key factors: (i) insufficient metabolic energy required to sustain the nitrogenase activity^3–5^; (ii) limited availability of different vitamins (*e.g*., cobalamin)^6^ and other micronutrients (*e.g*., Fe and Mo) required for the nitrogenase complex^7,8^; (iii) oxidized environments that irreversibly damage the nitrogenase enzyme^9–11^; and (iv) inhibitory (high) concentrations of dissolved inorganic nitrogen^12,13^.

Recent studies indicate that planktonic heterotrophic diazotrophs are highly diverse^14–16^, and fix N_2_ in environments characterized by one or more of the conditions considered adverse for diazotrophy. These environments include oxygenated waters^17–20^, aphotic (dark) oxygen-minimum zones^21–25^, and N-rich ecosystems^14,16,26–28^. Currently, the mechanisms explaining how heteroterotrophic diazotrophs cope with these unfavaroble conditions are not entirely clear. It was previously hypothesized that heterotrophic diazotrophs may benefit from the association with transparent exopolymer particles (TEP) as microenvironments that enable them to fix N_2_ even in unfavorable marine/freshwater regimes^17^. TEP are clear and sticky acidic polysaccharides, ranging in size from ~0.4 μm to 300 µm, and found at high concentrations across the aquatic environment^29–34^. The C:N ratio of TEP is often high and range from ~7 to 21 (average ~16:1) compared to the ~6.6:1 Redfield ratio^35–39^. TEP often act as a scaffold, holding together different organic and inorganic particles that form larger (>0.5 mm) aggregates^40–42^. These free-floating aggregates, also known as marine/river/lake “snow”, are ubiquitous throughout the aquatic environment^36,43–45^. Planktonic aggregates are heavily colonized by various microorganisms such as eukaryotic algae, fungi, bacteria, archaea and cyanobacteria^34,46–48^. Prokaryotic microorganisms that colonize aggregates solubilize and remineralize organic matter at high rates^49^ via a wide range of hydrolytic ecto-enzymes^50–52^. Such aggregates are therefore considered as “hotspots” for intense microbial activity^33,53,54^ and potentially also for N_2_ fixation by diazotrophs^16,17,19,34,55^.

Previous studies based the association of aggregates and heterotrophic diazotrophs on indirect links, including unspecific bacterial staining and statistical approaches^17,25,55–57^. In this study, we identified, for the first time, a direct link between heterotrophic N_2_ fixation and aggregates that comprise polysaccharides (such as TEP). To this end, we developed a new staining method that localizes heterotrophic diazotrophs by immunolabeling the nitrogenase enzyme, polysaccharides that form the aggregate matrix by Alcian blue and concanavalin-A staining, as well as total bacteria by DNA staining. This new staining approach, together with other analytical assays, was tested using a model heterotrophic diazotroph (*Vibrio natriegens*) and validated in a natural eutrophic N-rich estuary ecosystem.

## Materials and Methods

### Bacterial strains and experimental media

Monoculture experiments were performed using *V. natriegens* (ATCC 14048) as a model heterotrophic diazotroph^58,59^. Monocultures of *Escherichia coli* (ATCC 11303) were also used as a negative, non-diazotrophic control. *V. natriegens* and *E. coli* were cultivated in artificial brackish water supplemented with glucose (5 g L^−1^) and ammonium chloride (1.5 mg L^−1^ NH_4_Cl). Further details are provided in the Supporting Information (SI).

### Controlled laboratory experiments

A starter culture of *V. natriegens* (25–30 mL) was grown overnight to ~0.8–1.2 (OD_600 nm_) in a Luria-Bertani broth (LB) medium (LB, Merck Millipore, USA) with 1.5% NaCl. The cultures were then diluted to an early exponential growth phase (OD_600 nm_, 0.4–0.6) at 26 °C. The LB was removed after centrifugation (1500 g for 6 min) and *V. natriegens* bacteria were re-suspended in artificial brackish water (25 mL). *V. natriegens* cells were then transferred to sterile 1-L microcosm bottles with artificial brackish water in a ratio of 1:20 (vol:vol). The microcosm bottles were then supplemented with gum xanthan (GX, final concentration of 600 μg L^−1^) as an artificial polysaccharide and incubated either under aerobic or anaerobic conditions. Unamended microcosm bottles (without GX) were used as control. Three out of the four microcosms of each treatment were enriched with ^15^N_2_ and incubated for 48 h under dark conditions at 26 °C with gentle shaking. Bacterial abundance (BA) and bacterial production (BP) rates were measured at the conclusion of the incubation (detailed below). N_2_ fixation rates and immunolabeling of the nitrogenase protein were also determined at the conclusion of the experiment. Simultaneous experiments were carried out with the non-diazotrophic *E. coli* (*i.e*., without the nitrogenase enzyme) as a negative control. These experiments were performed to refute unspecific nitrogenase tagging.

### Field samplings in the Qishon estuary

Surface (~0.3 m) saline water was collected from the Qishon estuary (32°48′42.0″N, 35°02′00.7″E) during the winter (February 2018). The collected water was divided into four pre-cleaned (10% HCl and autoclaved) 1-L Nalgene bottles. Three bottles were enriched with ultra-pure ^15^N_2_ water (detailed below), while the forth bottle was used to measure the natural abundance of dissolved ^15^N_2_ (*i.e*., no isotope addition). All bottles were supplemented with 3-(3,4-dichlorophenyl)-1,1-dimethylurea (DCMU, final concentration of 100 nM, Sigma-Aldrich D2425) and covered with an aluminum foil to impair photosynthetic activity^18,20,34,60^. The microcosm bottles were incubated at ambient temperature in dark conditions for 48 h.

### Tagging active heterotrophic diazotrophs associated with artificial and natural aggregates

#### Visualizing V. natriegens monocultures associated with artificial TEP surrogates

**Figure 1:**
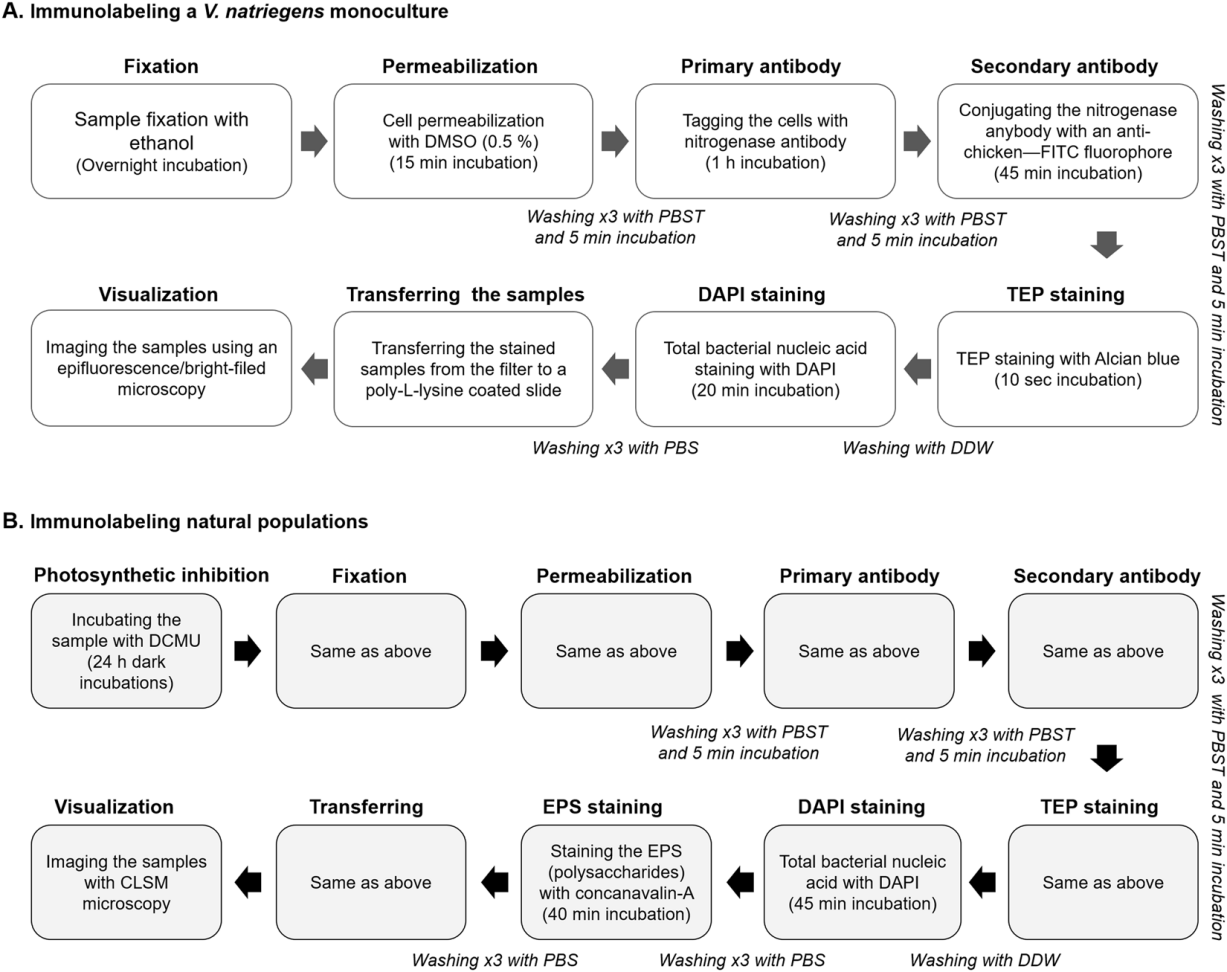
Overview of the immunolabeling staining approach used in this study for monocultures (**A**) and natural (**B**) populations capturing heterotrophic diazotrophs associated with bioaggregates.

Subsamples (3 mL) of *V. natriegens* or *E. coli* were collected at the end of the incubation and filtered through a 0.4 µm polycarbonate filter (GVS, Life Sciences, USA) using low vacuum pressure (<150 mbar) (Fig. 1A). Filters with bacteria were fixed overnight in chilled ethanol (5 mL), while residues were removed at the end of the incubation by a gentle filtration (<150 mbar). *V. natriegens* (or *E. coli*) cells were permeabilized by 1 mL dimethyl sulfoxide (DMSO, 0.5%, Merck Millipore 102952) for 15 min at room temperature and washed three times with 5 mL of phosphate buffered saline enriched with triton (PBST, 0.1% Triton X-100 in PBS, pH 7.2, Sigma Aldrich)^61^. Cells were then incubated for 5 min in PBST. An anti-NifH (Agrisera-AS01 021A, Sweden) solution (6 µg mL^−1^) that binds to the nitrogenase enzyme was freshly prepared. The anti-NifH solution was diluted in PBS-bovine albumin serum (PBS-BSA, 1 mg mL^−1^, Sigma Aldrich A2153) to minimize unspecific antibody binding. The cells were incubated for 1 h in the dark with the primary anti-NifH antibody to tag the nitrogenase enzyme. Filters with tagged cells were washed with PBST as described above before an anti-chicken antigen conjugated to a fluorescein isothiocyanate (FITC) fluorophore (6 μg mL^−1^, Thermo Fisher Scientific A-11039) was added and incubated in the dark for 45 min. This florescent secondary antibody was specifically linked to the primary antibody and used to visualize (spectra is detailed below) the presence of the nitrogenase enzyme. Any antibody residues were discarded with PBST as described above. The washing efficiency of the secondary antibody was tested to verify that the fluorescence resulted only from a successful connection between the antibodies (Fig. S1).

In the second stage, the immunolabeled samples were stained for TEP using an Alcian blue solution (4%) for 10 sec similarly to Bar-Zeev *et al*.^48^. The samples were then washed with 5 mL of double distilled water (DDW) to remove any residues of Alcian blue stain that were not adsorbed to the polysaccharides. In the third stage, the immunolabeled and TEP-stained samples were incubated with 4′,6-diamidino-2-phenylindole solution (DAPI, 250 µg mL^−1^, Thermo Fisher Scientific D1306) for 20 min to visualize all the bacterial cells^33^. The DAPI excess was removed with 5 mL of PBS.

Finally, each filter with the immunolabeled-stained sample was placed (face-down) for 3 min on a poly-L-lysine (P8290, Sigma Aldrich) coated microscope slide (detailed in the Supporting Information). The samples remained attached to the poly-L-lysine coating while the filter was gently removed. The attached cells and aggregates were covered with a cover slip (2 × 1 cm) and sealed with nail-polish to minimize dehydration. Each sample was examined with a Nikon Eclipse Ci epifluorescence microscope. TEP were visualized under a bright field light while fluorescence was used to identify total bacteria (DAPI; Ex 350 nm, Em 450 nm) and the immunolabeled nitrogenase enzyme (FITC; Ex 495 nm, Em 519 nm).

#### Localizing heterotrophic diazotrophs that colonize aggregates in aquatic environments

Water samples (5 mL) from the Qishon estuary were stained similarly to the protocol described above with a few modifications (Fig. 1B). Initially, the samples were incubated in the dark and supplemented with DCMU (100 nM) to hinder active phototrophic diazotrophs that may have been present in the ambient water^20,62,63^. The incubation time of the DAPI stain was increased to 45 min to ensure the diffusion of the dye into large aggregates (>0.5 mm). Polysaccharides were stained with the fluorescent lectin, concanavalin A (200 µg L^−1^, Ex 630 nm, Em 647 nm, Thermo Fisher Scientific C11252) for 40 min and washed with 5 mL of PBS. Stained samples were imaged with a Zeiss confocal laser scanning microscope, CLSM (LSM 510 Meta) equipped with a 405 nm diode and 488 nm argon as well as 633 nm helium-neon lasers. Concurrently, the samples were stained with Alcian blue and imaged via bright field mode to identify TEP (Fig. S2). Heterotrophic bacteria were distinguished from phototrophic cyanobacteria by subtraction of the auto-fluorescence of phycoerythrin (Ex 490 nm, Em 580 nm) as an indicative pigment (Fig. S3).

### Analytical approaches

#### N_2_ fixation rate

Measurements were done as described by Mohr *et al*.^64^. Briefly, an enriched ^15^N_2_ medium was prepared by injecting ^15^N_2_ gas (99%, Cambridge Isotopes) into pre-filtered (0.2 µm) artificial medium at a 1:100 (vol:vol) ratio. The enriched stock was vigorously shaken to completely dissolve the ^15^N_2_ gas bubble, and then added to the experimental bottles (0.5% and 5% of monoculture media and Qishon sample volume, respectively^18^). This procedure assured adequate equilibration of the ^15^N_2_ with the media/estuary water. Following two days of dark incubation, the samples were filtered through pre-combusted 25-mm GF/F (450 °C, 4.5 h) and dried overnight at 60 °C. The samples were analyzed on a Thermo-Finningan Delta Plus XP isotope ratio mass spectrometer (IRMS) interfaced to a CE Instruments NC2500 elemental analyzer. A standard curve to determine N mass was done with each sample run. The detection limit was 0.02 nmol N L^−1^ d^−1^.

#### Bacterial production (BP)

Rates were measured using the incorporated [4,5-^3^H]-leucine method (Amersham, specific activity of 160 Ci nmol^−1^) according to Simon *et al*.^65^. Samples were incubated with 2 nmol leucine L^−1^ (final concentration) in rotation for 3 h at ~25 °C and dark conditions. Incorporation of leucine was converted to carbon by a conservative factor of 3.1 kg C mol^−1^ with an isotope dilution of 2.0^66^.

#### Bacterial abundance (BA)

Collected samples (1.7 mL) were fixed with 0.2% glutaraldehyde (Sigma Aldrich) for 10 min and stored at −80 °C. Prior to counting, samples were fast-thawed at 26 °C, and bioaggregates were dismantled using 25 mM of EDTA and sonication. Samples were stained with 0.5 nM of SYBR® Green II RNA Gel Stain (Thermo Fisher Scientific S7564) in the dark for 15 min^67^. Subsamples (150 µL) were analyzed with an Attune-Next acoustic focusing flow cytometer (Applied Biosystems) equipped with a syringe-based fluidic system at 408 and 488 nm wavelengths at a flow rate of 25 µL min^−1^ ^68^. Beads (nominal size 0.93 µm) (Polysciences) were used as a size standard.

### Statistical analysis

Statistical analysis was performed using an Excel add-in XLSTAT 2018 software. The differences between treatments (Control, +GX, under anaerobic and aerobic conditions) were evaluated using ANOVA following Fisher post-hoc test with a confidence level of 95%. For the Qishon microcosms, the differences between the ambient (unamended) and the +GX waters were evaluated using a t-test with a confidence level of 95%.

## Results and Discussion

### Immunolocalization and N_2_ fixation of *V. natriegens* diazotrophs associated with TEP

**Figure 2:**
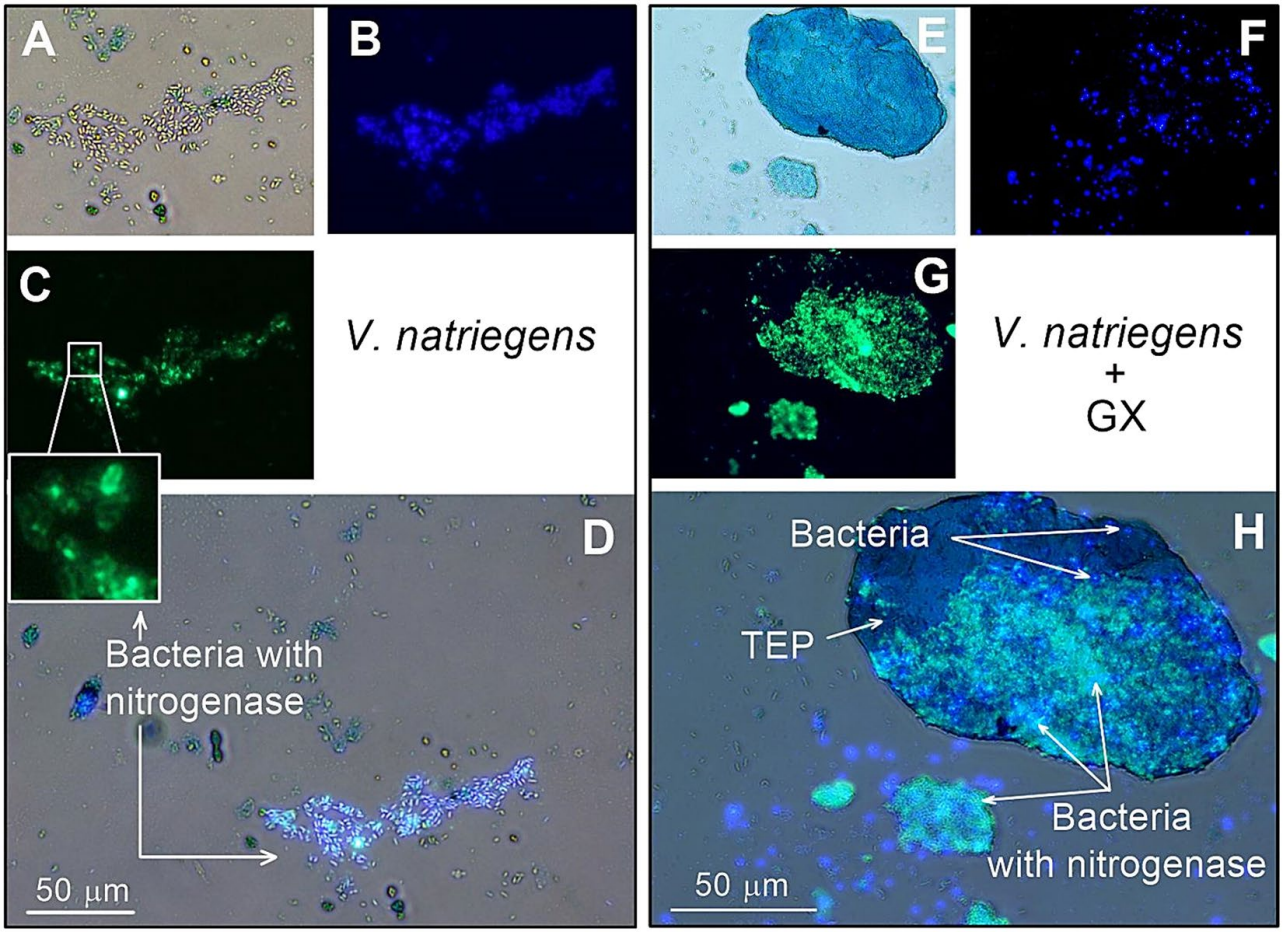
Visualization of *V. natriegens* as a model heterotrophic diazotroph, TEP and total bacteria using the newly develop triple-staining method. Images were captured under anaerobic conditions with media only (**A**–**D**) or following the addition of GX (**E**–**H**). TEP were stained by Alcian blue (**A**,**E**), while total bacteria were stained with DAPI **(B**,**F**), and the nitrogenase enzyme was tagged by immunolabeling (**C**,**G**). Images were stacked and superimposed using an ImageJ software (**D**,**H**).

**Figure 3:**
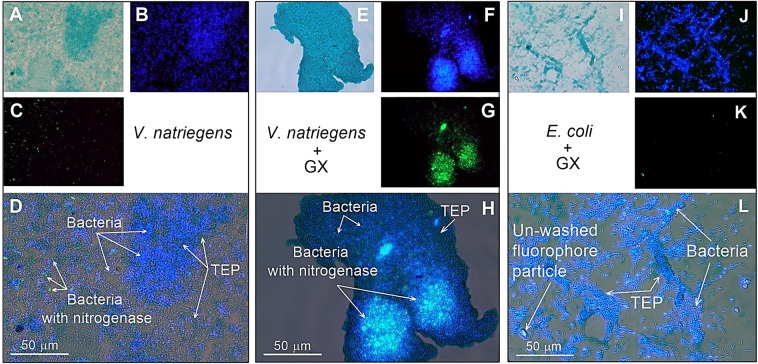
Images of *V. natriegens* and *E. coli* under aerobic conditions, with or without GX, captured by epifluorescence microscopy. TEP stained with alcian blue (**A**,**E**,**I**; light blue); total bacteria stained with DAPI (**B**,**F**,**J**; blue); active diazotrophs tagged by nitrogenase immunolabeling (**C**,**G**,**K**; green). Superimposed images were done using ImageJ software (**D**,**H**,**L**).

Planktonic heterotrophic diazotrophs such as *V. natriegens* are ubiquitous facultative anaerobes^58^ that can be cultivated with simple carbon molecules such as glucose or sucrose^59,69^. N_2_ fixation by *V. natriegens* can be hindered by low availability of organic carbon sources and/or high concentrations of dissolved inorganic nitrogen^1,70,71^. Previous reports have suggested that heterotrophic diazotrophs associated with TEP may explain N_2_ fixation rates in aquatic environments with adverse conditions for diazotrophy^16,17^. Yet, no direct link was previously found between heterotrophic diazotrophs and aquatic aggregates. Our newly developed staining method is the first to provide a direct link between active heterotrophic diazotrophs and aggregates comprising polysaccharides such as TEP (Figs 2 and 3).

Under anaerobic conditions with no addition of GX (*i.e*., artificial TEP), the nitrogenase enzyme of *V. natriegens* was captured within most of the cells using our new staining approach (Fig. 2A–C). Concomitant measurements of N_2_ fixation and BP rates were normalized to bacterial cells (2 to 5.7 × 10^10^ cells L^−1^), resulting in specific rates per cell. Specific N_2_ fixation ranged from 1.2 to 3.9 × 10^−4^ fg N cell^−1^ d^−1^ and specific BP ranged from ~1.4 to 7.1 fg C d^−1^ (Table 1, n = 9). These measurements, together with the immunolabeling technique (Fig. 2C), indicate that under anaerobic conditions *V. natriegens* were active, synthesizing the nitrogenase enzyme and fixing N_2_.

**Table 1:** Average N_2_ fixation, bacterial production (BP) and bacterial abundance (BA) of *V. natriegens* under different aeration conditions and with or without the addition of GX.

Aeration	Treatment	N_2_ fixation (nmole N L^−1^ d^−1^)	BP (µg C L^−1^ d^−1^)	BA (×10^10^ Cells L^−1^)	Specific N_2_ fixation (×10^−4^ fg N Cell^−1^ d^−1^)	Specific BP (fg C Cell^−1^ d^−1^)
Anaerobic	Control	3.8 ± 1.5^a,b^	94.5 ± 44.2^a^	2.5 ± 0.4^a^	2.1 ± 0.8^a^	3.8 ± 1.5^a^
	GX	5.9 ± 2.1^b,c^	68.4 ± 33.0^b^	3.0 ± 1.1^a^	2.8 ± 1^a^	1.9 ± 1.1^b^
Aerobic	Control	3.9 ± 1.0^a^	140.6 ± 36.5^c^	7.1 ± 2.5^b^	0.7 ± 0.2^b^	2.0 ± 0.5^b^
	GX	4.2 ± 0.5^a,b^	130.9 ± 25.5^c^	7.4 ± 1.7^b^	0.8 ± 0.1^b^	1.8 ± 0.4^c^

Specific N_2_ fixation rates and specific bacterial production rates were calculated by normalizing N_2_ fixation and BP to BA. ^*^The superscripts letters (a,b,c) indicate a significant difference for mean values additions (one-way ANOVA and a Fisher LSD post hoc test, P < 0.05).

Incubation of *V. natriegens* with GX for 48 h under anaerobic conditions resulted in the formation of aggregates that comprised TEP (Fig. 2E). Most of the active *V. natriegens* cells (with the nitrogenase enzyme) were found to be associated with the TEP-aggregates forming dense diazotrophic clusters (Fig. 2G,H). In addition, GX amendments increased N_2_ fixation rates (55%) and BA (20%), while BP was reduced (28%) relative to the control experiments (Table 1). Specific N_2_ fixation rates were also higher (33%) following incubation with GX, while BP rates per cell remained significantly lower than the control (Table 1). BP often indicates on the assimilation rates of organic carbon into bacterial biomass^34^. Thus, we suggest that low BP rates and increased N_2_ fixation rates (compared to the control) indicated that additional carbon source following polysaccharide hydrolysis^56,72^ was mostly utilized to support nitrogenase activity rather than bacterial growth.

Additional experiments under aerobic (O_2_-saturated) conditions with *V. natriegens* were also carried out in media only (control) or following the addition of GX (Fig. 3, Table 1). Under control conditions (no GX addition) only sparse extracellular polymeric substances (consisting of polysaccharides) were found to be stained with Alcian blue although bacteria were highly abundant (Fig. 3A,B). Planktonic *V. natriegens* cells were sporadically captured with the nitrogenase enzyme (Fig. 3C,D). Under these conditions N_2_ fixation rates by *V. natriegens* remained similar to those measured under anaerobic conditions (Table 1). Concomitant BP rates and BA values were significantly higher (50% and 280%, respectively) than those measured under anaerobic conditions (Table 1). Conversely, cell specific BP and N_2_ fixation rates were substantially lower (47% and 67%, respectively) compared to the anaerobic conditions (Table 1). As facultative anaerobes, *V. natriegens* gain more energy-rich molecules under aerobic conditions (*e.g*., ATP and NADPH) compared to anaerobic conditions, thus may enhance bacterial growth rates (measured as increased BA). However, under aerobic conditions it is likely that *V. natriegens* allocated metabolic energy to protect the nitrogenase enzyme from O_2_ damages^10,73^, leading to lower carbon assimilation (BP) per cell compared to the anaerobic rates, yet measurable N_2_ fixation (Table 1).

Under aerobic conditions and following incubation with GX, we rarely detected planktonic cells that translated the nitrogenase enzyme (Fig. 3E–H). Instead, most *V. natriegens* cells with the nitrogenase enzyme formed dense clusters associated with TEP (Fig. 3H). Concomitant measurements of N_2_ fixation, BP and BA were all comparable to the values found in the aerobic control. Similar results were also found for the cell-specific N_2_ fixation and BP rates (Table 1). Nonetheless, compared to the anaerobic conditions, BP and BA were ~2 fold higher, while N_2_ fixation rates remained lower (29%) following the addition of GX (Table 1). We surmise that the artificial TEP (GX) aggregates formed in these experiments did not provide additional advantage to N_2_ fixation under aerobic conditions. We suggest that since these aggregates were overall small (<300 µm), O_2_ concentrations within the particles were similar to the surrounding environment. Nonetheless, it is possible that larger aggregates may provide conditions with reduced O_2_ concentrations that benefit heterotrophic diazotrophs as previously reported by Klawonn *et al*.^74^.

In addition to the above, *E. coli* as non-diazotrophic bacteria were grown under aerobic conditions with or without GX for 48 h and used as a negative control (Fig. 3I–L). These experiments were performed to estimate whether unspecific tagging of the primary and secondary antibodies to the cells or TEP occurred. As expected, following the staining of *E. coli* cells, no green florescence was visible (Fig. 3K).

### Localizing heterotrophic diazotrophs associated with natural aggregates using the Qishon Estuary as a case study

The Qishon estuary system is defined as a hyper-eutrophic environment that flow into the Haifa Bay, southeastern Mediterranean Sea^34,75^. Nutrient concentrations during the sampling period in the Qishon estuary were an order of magnitude higher (TN 5.8 mg L^−1^, TP 0.4 mg L^−1^, TOC 72 mg L^−1^) than the southeastern Mediterranean Sea^76,77^. During the winter sampling, total BA was 3.5 × 10^9^ cells L^−1^, while BP rates were 80.2 µg C L^−1^ d^−1^. In addition, heterotrophic N_2_ fixation rates were 0.96 nmol N L^−1^ d^−1^ following dark incubation with DCMU which have likely impaired the activity of phototrophs^18,34^. These N_2_ fixation rates were ~2–3 fold higher than previous reports from the southeastern Mediterranean Sea, despite the high concentrations of ambient TN^58,77–79^ that should suppress diazotrophy^13^.

**Figure 4:**
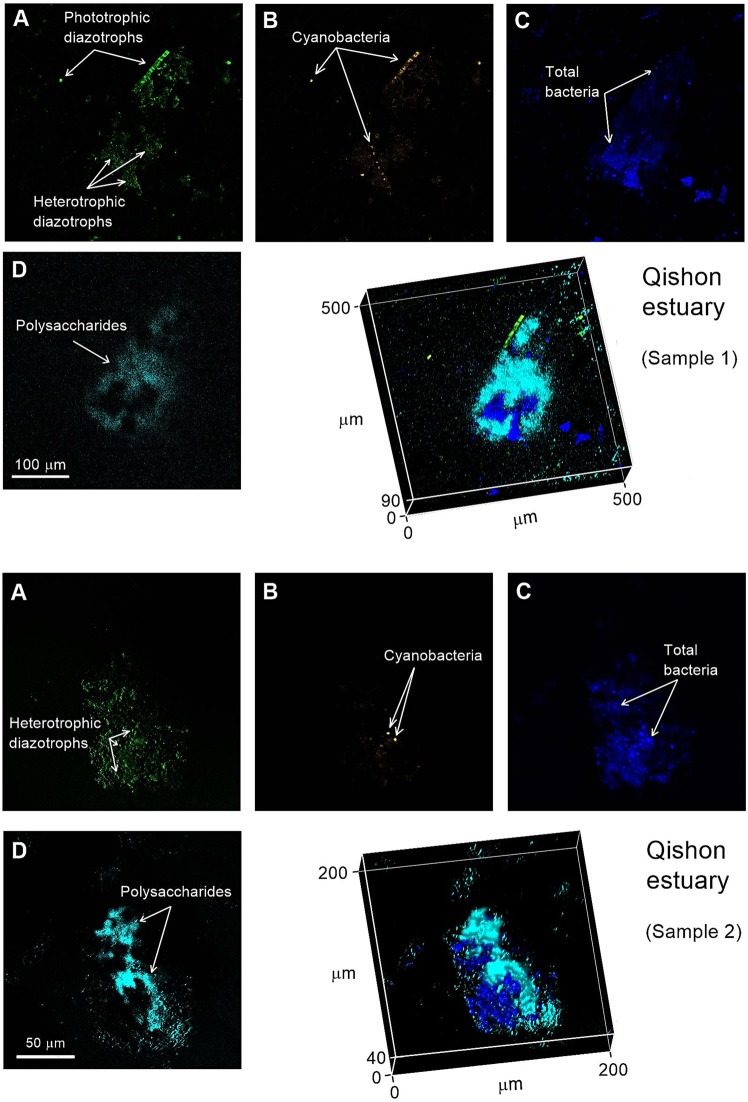
Two examples of natural diazotrophs associated with aggregates collected from the Qishon estuary and captured by CLSM. Diazotrophs that have synthesized the nitrogenase enzyme were tagged by immunolabeling (**A**, green), cyanobacteria were identified with phycoerythrin (**B**, orange), total bacteria with DAPI (**C**, blue), and polysaccharides were stained with Con A (**D**, light blue). Superimposed three-dimension images of the different stains were made using Zen software.

In aquatic environment such as the Qishon estuary, diazotrophs often comprise a diverse community which include archaea and heterotrophic bacteria^14,16^, as well as phototrophs in the form of filamentous and/or unicellular cyanobacteria^13,71^. In agreement with these reports, we found that these aggregates comprised complex microbial communities, including various prokaryotic microorganisms (identified via DAPI staining), cyanobacteria (characterized by the auto-fluorescence of phycoerythrin), as well as active diazotrophs (localized by nitrogenase immunolabeling). These complex microbial communities were embedded in a polysaccharide matrix (detected by a fluorescent lectin) and colonized the entire aggregate with no specific pattern (Fig. 4).

Immunolabeling the nitrogenase enzyme is not specific to heterotrophic diazotrophs and may tag phototrophic diazotrophs as well (Fig. 4A, top panel). Differentiating and localizing active heterotrophic diazotrophs was achieved via the following: (i) Suppression of phototrophic diazotrophs by incubating the samples in the dark for 48 h with a photosynthetic inhibitor (DCMU)^60^. We surmised that these conditions would reduce the activity and biosynthesis of the nitrogenase by phototrophic diazotrophs due to the lack of metabolic energy^18,20,56^. (ii) Matching images of the phycoerythrin pigment auto-fluorescence were captured from each sample to identify any cyanobacteria that could potentially fix N_2_ (Fig. 4B, top panel). (iii) Overlapping images of cyanobacteria (orange) by immunolabeled (green) and total microorganisms (blue) enabled the specific identification of heterotrophic bacteria that translated the nitrogenase protein. (iv) The spatial location of these heterotrophic diazotrophs on the aggregate was attained by superimposing the previous stack with images of fluorescent polysaccharides (light blue) (Fig. 4D). Applying this new method to natural water samples together with ^15^N_2_ assays indicated that planktonic aggregates from the Qishon estuary were colonized by heterotrophic diazotrophs that were actively synthesizing the nitrogenase enzyme.

## Conclusions

Recent studies suggested that heterotrophic diazotrophs are more widely distributed than previously thought, fixing N_2_ in “unfavorable” aquatic environments. These studies suggested that in such environments N_2_ fixation is mainly performed by heterotrophic diazotrophs that colonize bioaggregates comprising polysaccharides such as TEP. However, to date, no direct link between heterotrophic diazotrophy and bioaggregates was ever established and only indirect evidence, namely correlations between TEP and N_2_ fixation rates, has been shown. Here, we introduce a newly developed staining approach that directly link active heterotrophic diazotrophs (as well as phototrophic diazotrophs) to aggregates. We show that this new method provides specific spatial localization of heterotrophic diazotrophs on artificial and natural aggregates comprising polysaccharides. Nonetheless, the specific N_2_ fixation rates by diazotrophs associated with aggregates cannot be estimated using this approach. To date, it is possible to compliment this method with size fractionation approaches and distinguish between N_2_ fixation rates by planktonic diazotrophs to those associated with aggregates of various sizes. We stress that future research should focus on developing complimentary visualization techniques such as NanoSIMS that may localize diazotrophs on aggregates while providing cell specific N_2_ fixation rates at the same time. These novel approaches will provide new insights on the different roles of aggregates in supporting heterotrophic N_2_ fixation. Moreover, insights gained by these visualization approaches may have a broader significance in future research of N_2_ fixation and the aquatic nitrogen cycle, including environments in which diazotrophy has been traditionally overlooked.

## Supplementary information

Direct Detection of Heterotrophic Diazotrophs Associated with Planktonic Aggregates

## Data Availability

All data measured and analyzed during this study are included in the manuscript.
