# Supplementary material for: Direct Detection of Heterotrophic Diazotrophs Associated with Planktonic Aggregates
Source: Sci Rep. 2019 Jun 26;9:9288. doi: 10.1038/s41598-019-45505-4 (PMC6594930; doi:10.1038/s41598-019-45505-4)
Supplement: Supplementary file 1 — Direct Detection of Heterotrophic Diazotrophs Associated with Planktonic Aggregates [file 41598_2019_45505_MOESM1_ESM.docx]

**Supporting Information**

**Direct Detection of Heterotrophic Diazotrophs Associated with Planktonic Aggregates**

Eyal Geisler^1,2^, Anne Bogler^1^, Eyal Rahav^2*^ and Edo Bar-Zeev^1*^

1. Zuckerberg Institute for Water Research (ZIWR), The Jacob Blaustein Institutes for Desert Research (BIDR) Ben-Gurion University of the Negev, Sede Boqer Campus, 84990, Israel
2. Israel Oceanographic and Limnological Research, National Institute of Oceanography, Haifa, 8030, Israel

**Corresponding authors:**

Edo Bar-Zeev; [barzeeve@bgu.ac.il](mailto:barzeeve@bgu.ac.il)

Eyal Rahav; [eyal.rahav@ocean.org.il](mailto:eyal.rahav@ocean.org.il)

## Materials and Methods

***Vibrio natriegens* artificial media**

Artificial brackish water was made fresh by dissolving 15 g of sea salt (Advanced Pro Formula Salt, Royal Nature) in autoclaved Double distilled water (DDW) (1-L) and filtered through filter paper (Macherey-Nagel). Sodium bicarbonate NaHCO_3_ was added (15 mg L^-1^) to maintain a pH of 7–7.2. Vitamin (0.75 µM cobalamin, 4 µM biotin and 0.8 µM thiamine HCl) and trace metal (4.8 mM [FeCl]x6H_2_O, 17 µM Na_2_EDTAx2H_2_O, 40 µM [CuSO_4_]x5H_2_O, 28 µM [NaMoO_4_]x2H_2_O, 76 µM [ZnSO_4_]x7H_2_O, 42 µM [CoCl_2_]x6H_2_O, 1 mM [MnCl_2_]x4H_2_O) solutions were filtered through a 0.22-µm filter (Millex SLGV033RS) and added to the sterile media.

Concurrently, gum xanthan (GX, 20 mg) (Sigma Aldrich G1253) was added to MilliQ water (200 ml) and left for 15 min to swell. GX was then ground using homogenizer (HSIANGTAI HG-300) according to Passow and Alldredge 1995 ^1^. The GX solution was left under a UV light (Bioforce Nanoscience, model UV.TC:220) for 1 h to minimize contamination. Analysis of the GX solution indicated that GX was comprised of 22.4 mg L^-1^ of total organic carbon (TOC) and 0.5 mg L^-1^ total nitrogen (TN), resulting in a C:N ratio of ~45.

**Preparing poly-L-lysine-coated slides**

The slides were rinsed with HPLC grade acetone (100%) and dried with N_2_ gas, followed by rinsing with 100% ethanol. A final rinse with DDW was done to remove any ethanol or acetone residues. The cleaned slides were exposed to UV light for 20 min to remove any organic material that was still attached to the slide surface. At the same time, a poly-L-lysine work solution was prepared by adding MilliQ water to a poly-L-lysine stock solution (Sigma Aldrich P8920) in a 1:1 ratio (vol:vol). The poly-L-lysine work solution (250 µL) was drop-cast on the sterilized slides and left for 5 min. Poly-L-lysine residues were washed from the slide with DDW and dried overnight at room temperature (~25°C) in a close petri-dish.

**Searching for any traces of unspecific staining by the secondary antibody in monocultures and natural samples**

The washing efficiency of the secondary antibody that was conjugated to the FITC fluorophore was tested to verify that the green fluorescence resulted only from the link with the primary antibody (*i.e.,* excluding unspecific fluorescence). To this end, we performed the same staining protocol as described for the monoculture\natural populations, but without labeling them with the primary antibody. Our results indicate that no green fluorescence was found following the staining of the samples with the second antibody (Figure S1). Therefore, we surmise that the link with the primary antibody is a crucial step for successful nitrogenase labeling. In addition, we deduce that the stain was not adsorbed to the organic matter in the samples.

**
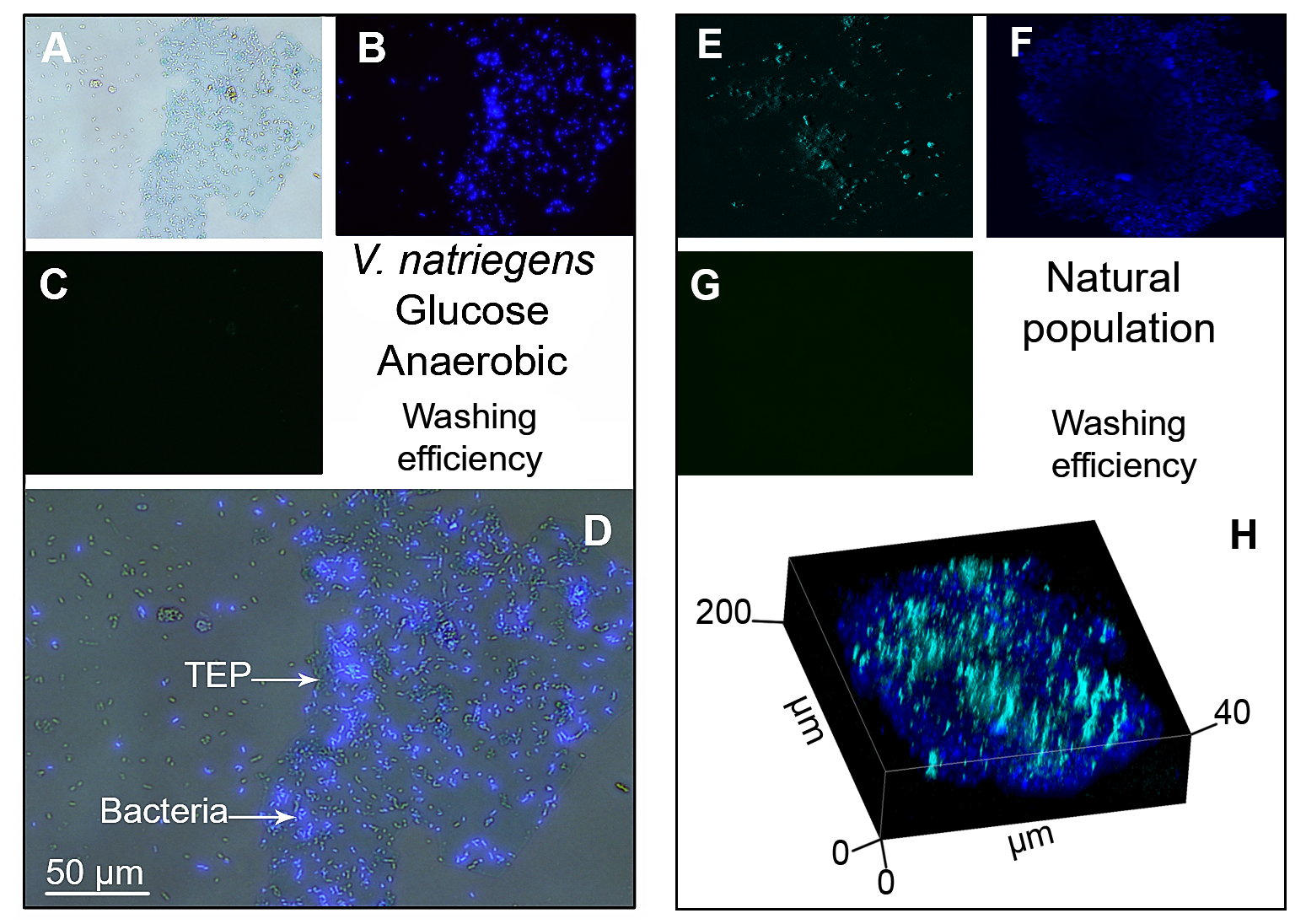
**

**Figure S1.** Washing efficiency of the secondary antibody from a *V. natriegens* monoculture (left panel) and a natural sample (right panel). *V. natriegens* were stained for (A) TEP with Alcian blue, (B) total bacteria stained with DAPI, (C) secondary antibody only, and (D) superimposed image of the different stains. The natural sample was immunolabeled with the secondary antibody only (E), stained for total bacteria with DAPI (F), and polysaccharides with Con A (G); the 3D micrograph that was compiled from the previous images (H).

**Visualizing TEP and polysaccharides in the Qishon ecosystem by Alcian blue and concanavalin A**

TEP are defined as acidic polysaccharides since they are stained with Alcian blue ^1^. However, Alcian blue cannot be visualized and scanned by CLSM to produce a 3D image since it does not comprise a fluorescence element. Therefore, natural polysaccharide samples were labeled using concanavalin A (Con A, Ex 630 nm, Em 647 nm), a fluorescent lectin that stains various polysaccharides ^2^. In order to verify that TEP formed the polysaccharide matrix, we also stained the samples with Alcian blue. These samples were then imaged using CLSM in bright field mode (Figure S2 A). Matching images were then captured by the CLSM using the fluorescence of Con A (Fig. S3 B). These results indicate that TEP formed the bioaggregate matrix that was collected from the Qishon ecosystem samples.


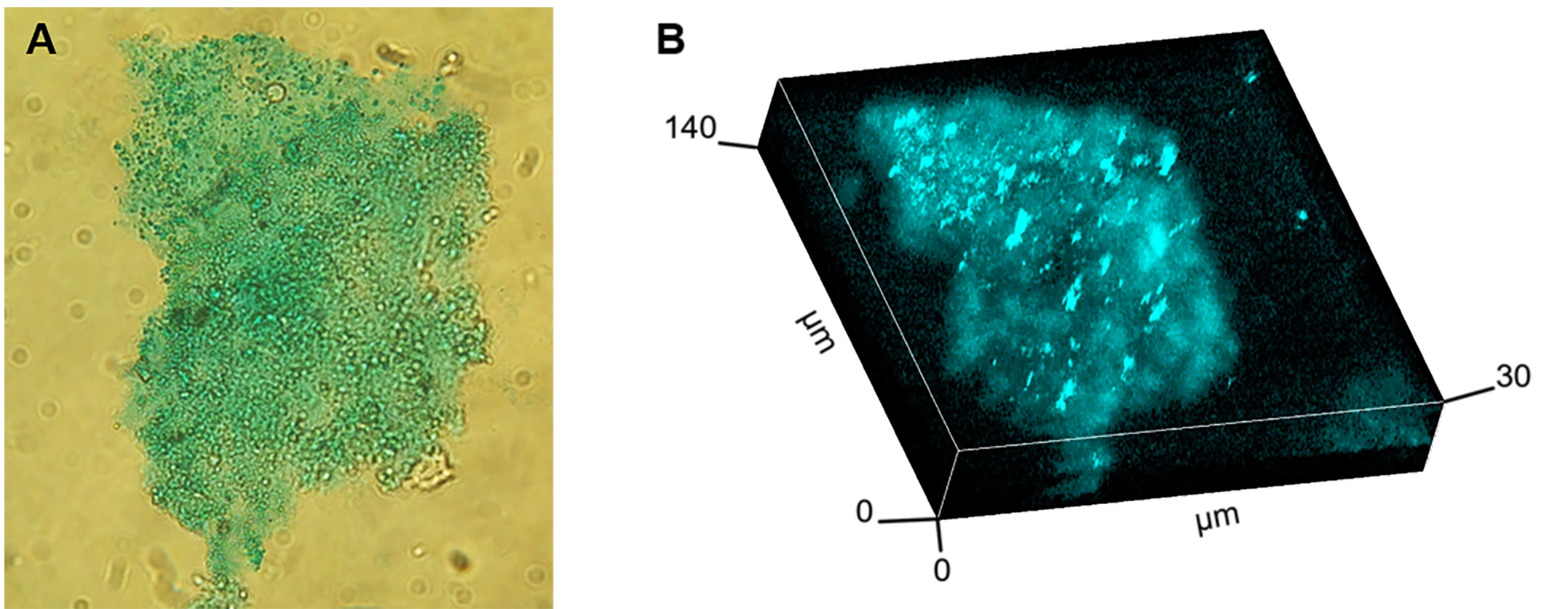


**Figure S2.** Visualization of TEP under (A) bright field microscopy and (B) CLSM.

**Identifying cyanobacterial cells by the auto-fluorescence of the phycoerythrin pigment**

Phycoerythrin fluorescence **(**Ex 490 nm, Em 580 nm), an exclusive pigment for cyanobacteria, was examined in order to detect the auto-fluorescence of potentially diazotrophic photo-autotrophs. A culture of *Nostocaceae* cyanobacteria was used to adjust the CLSM-specific settings needed to detect the phycoerythrin pigment (Figure S3). This pre-setup included measuring the most suitable excitation and emission spectra, as well as the power and gain of the argon laser (Ex of 488 nm), to detect phycoerythrin. This definition setup was then used for all the environmental samples. The *Nostocaceae* culture was isolated from Abronah, Israel in October 2017 (29°40'21.8"N, 35°00'46.8E) and grown in a mineral-free medium of nitrogen under a light regime at 25 ºC.


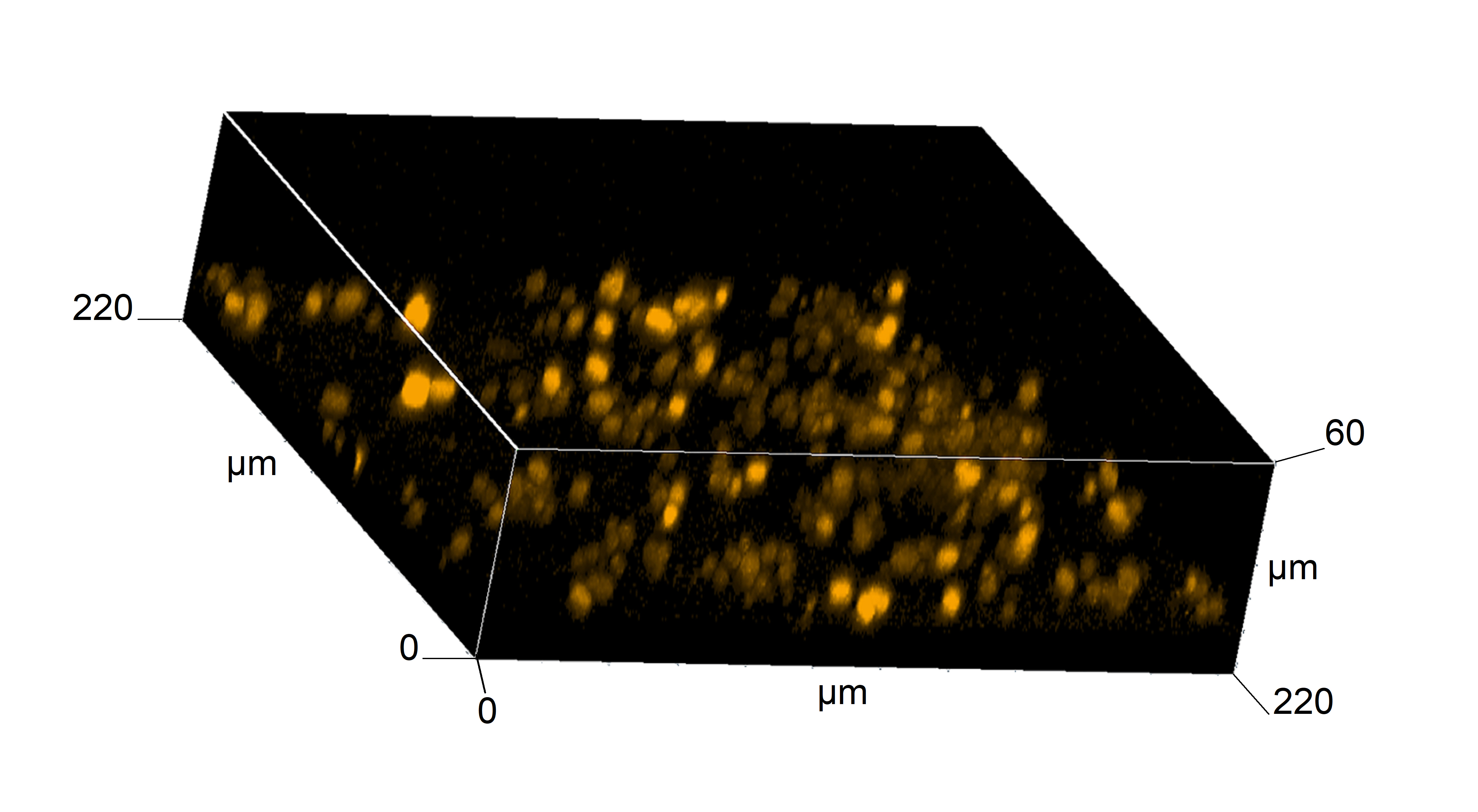


**Figure S3.** Representative 3D image of *Nostocaceae* cyanobacteria visualized via the auto-fluorescence of the Phycoerythrin pigment (Ex 490 nm, Em 580 nm) captured with CLSM using an argon laser with 488 nm.

**References**

1. Passow, U. & Alldredge, A. L. A dye-binding assay for the spectrophotometric measurement of transparent exopolymer particles (TEP). *Limnol. Oceanogr.* **40,** 1326–1335 (1995).

2. Bar-Zeev, E., Berman-Frank, I., Girshevitz, O. & Berman, T. Revised paradigm of aquatic biofilm formation facilitated by microgel transparent exopolymer particles. *Proc. Natl. Acad. Sci. U. S. A.* **109,** (2012).
